# From therapeutic antibodies to immune complex vaccines

**DOI:** 10.1038/s41541-018-0095-z

**Published:** 2019-01-17

**Authors:** Xuan-Yi Wang, Bin Wang, Yu-Mei Wen

**Affiliations:** 1 Key Laboratory of Medical Molecular Virology of MoE & MoH, Shanghai Medical College, Fudan University, 200032 Shanghai, China; 2 Institutes of Biomedical Sciences, Shanghai Medical College, Fudan University, 200032 Shanghai, China

## Abstract

In recent years, therapeutic monoclonal antibodies have made impressive progress, providing great benefit by successfully treating malignant and chronic inflammatory diseases. Monoclonal antibodies with broadly neutralizing effects against specific antigens, or that target specific immune regulators, manifest therapeutic effects via their Fab fragment specificities. Subsequently therapeutic efficacy is mediated mostly by interactions of the Fc fragments of the antibodies with their receptors (FcR) displayed on cells of the immune system. These interactions can trigger a series of immunoregulatory responses, involving both innate and adaptive immune systems and including cross-presentation of antigens, activation of CD_8_^+^ T cells and CD_4_^+^ T cells, phagocytosis, complement-mediated antibody-dependent cellular cytotoxicity (ADCC) and complement-dependent cytotoxicity (CDC). The nature of the triggered effector functions of the antibodies is markedly affected by the glycosylation patterns of the Fc fragments. These can cause differences in the conformation of the heavy chains of antibodies, with resultant changes in antibody binding affinity and activation of the complement system. Studies of the Fc glycosylation profiles together with the associated Fc effector functions and FcR/CR interactions promoted interest and progress in engineering therapeutic antibodies. Furthermore, because antigen–antibody immune complexes (ICs) have shown similar actions, in addition to certain novel immunoregulatory mechanisms that also reshape immune responses, the properties of ICs are being explored in new approaches for prevention and therapy of diseases. In this review, both basic studies and experimental/clinical applications of ICs leading to the development of preventive and therapeutic vaccines are presented.

## Introduction

Antibodies have been used for >100 years as effective therapies for infectious diseases. In the 1890s, pioneering studies from the laboratory of Robert Koch demonstrated that administration of sheep antiserum against diphtheria toxin to a girl dying from diphtheria led to her rapid recovery and ultimate survival.^1^ Later, more patients were treated with antibody therapy using horse antiserum against diphtheria toxin by von Behring and Kitasato, who were awarded the Nobel Prize. Since then, antimicrobial antibodies have also been used for the treatment of other infectious diseases, including bacterial pneumonia, staphylococcus infections, and septicemia.

Since the discovery of toxoid vaccines for prevention of tetanus and diphtheria, and the success of treating infections with antimicrobial drugs and antibiotics, the use of antibody therapies has been largely replaced by new and efficient therapies. To date, antitoxins against tetanus, and botulism, and pooled anti-rabies antibodies are still in use for prevention and treatment of the corresponding diseases. Pooled hyper-immune immunoglobulins for hepatitis B are recommended as passive immunization in combination with preventive vaccination for blocking perinatal transmission. Polyclonal immunoglobulins for hepatitis A and measles are used in people at risk or under emergency exposure. Respiratory syncytial virus (RSV) antibodies are used in infants born with low body weight, who may undergo a life-threatening episode of infection.

Development of murine monoclonal antibodies led to renewed interests in antibody therapy. However, low efficacy and the development of a human anti‐murine antibody (HAMA) response in patients has hampered the general use of these antibodies in clinics. During the last two decades, new technologies for generating mouse/human chimeric monoclonal antibodies and humanized monoclonal antibodies have resulted in more successful clinical antibody applications. Interest in antibody therapies has been further stimulated by the development of broadly neutralizing monoclonal antibodies against infections and monoclonal antibodies that target immune checkpoints for treatment of inflammation and immune disorders. Furthermore, rapid developments from studies on antibody structures and functions, from genetic engineering technology for mass production of proteins and from novel methods of applying therapeutic antibodies have further boosted interest. Studies of the immune mechanisms initiated in cells by Fc–FcR interactions, have resulted in perception of the immune regulatory roles of antigen–antibody immune complex (IC) as a double-edged sword being revisited and studied in more detail. Despite their potential for pathological effects, ICs have been explored as preventive in addition to therapeutic vaccines, first in poultry breeding, and later in human diseases. This review summarizes the background, the mechanistic studies on Fc–FcR functions, the translational research on Fc–FcR and the prospects of IC-based vaccines.

## Renewed interest in antibody therapy

The development of technology for generating murine monoclonal antibodies against antigens created a land mark for renewed interest in antibody therapy. Monoclonal antibodies surpass polyclonal immunoglobulins in several respects; they target identified epitopes, with higher specificity and potency, usually with higher efficacy and reduced side effects in clinical application and can be produced at a large scale. This superiority is exemplified by the fact that the polyclonal immunoglobulins that are licensed for prevention of RSV in high-risk infants born with low body weight have been officially replaced by monoclonal antibody.^2^

In early use as immunotherapies, rodent-derived monoclonal antibodies were relatively inefficient in human hosts. Most importantly, because mouse proteins are foreign to the human immune system, a human anti-mouse antibody (HAMA) response is elicited resulting in a rapid clearance of the mouse antibody and adverse reactions.^3,4^ Additionally, the Fc fragments of murine monoclonal antibodies are relatively inefficient in engaging in antibody-dependent cellular cytotoxicity (ADCC) and complement-dependent cytotoxicity (CDC), which are critical for immunological therapeutic effects. To overcome these disadvantages, engineered antibodies have been developed via multiple approaches. For example, to reduce immunogenicity of therapeutic mouse monoclonal antibodies, either the mouse Fc fragment or the whole antibody constant regions (CH1–CH3) were replaced with the human counterparts by means of genetic manipulations, whereas the mouse Fab or Fv (VH-VL) fragments retained the original epitope specificity.^5^ By these means, the immunogenicity to the human immune system is reduced by ≥70%.^6^ The first chimeric human–mouse monoclonal antibody, rituximab, was approved by Federal Drug Administration (FDA) in 1997.^7^ An alternative approach for production of chimeric human–animal antibodies is by using a humanized-rodent such as OmniRat that carries a chimeric human/rat IgH locus and a fully human Ig*κ* or Ig*λ* locus.^8^ In addition, fully human monoclonal antibodies can be produced after fusion of peripheral blood lymphocytes from immunized individuals, or immune B cells obtained at a disease recovery period, with human lymphoblastoid or lymphoma cell lines (human hybridomas).^9^ These important technologies have all contributed to facilitate the development of antibody therapy.

With new technologies in engineering antibodies, the application of therapeutic monoclonal antibodies has been extensively adopted in several medical fields.^10^ This is best shown in their use for treating cases of autoimmune disease and cancer patients. Immune checkpoint blockade has evolved as a new anticancer therapy. Checkpoints inhibitors are used to reactivate the exhausted tumor-specific T cells and restore cancer immune surveillance.^11^ Monoclonal antibody that target one of the immune checkpoints, anti-programmed death protein-1 (aPD-1) has demonstrated impressive benefit in some cancer patients.^12–14^ However, this approach is not effective in all patients. A recent article revealed that an in vivo tumor-associated macrophage pathway was associated with resistance to aPD-1 treatment,^14^ indicating that modification of Fc–FcγR interaction might improve the therapeutic efficacy of aPD-1. Other anticancer antibodies targeting specific proteins are under development or in clinical trials.^15^ The first therapeutic antibody for the treatment of inflammatory diseases was infliximab (Remicade; Centocor/Merck), in 1998, for the treatment of Crohn’s disease. This chimeric antibody binds both soluble and membrane-associated tumor necrosis factor (TNF).^16^ Later, additional TNF antagonists have been approved by FDA for human use. Enhancing effectiveness, prolonging half-life in vivo, and developing biphasic therapeutic monoclonal antibodies, which may be broadly applicable to different combinations of target antigens are important objectives to be pursued. An elegant review has summarized the development and application of monoclonal antibodies for the treatment of autoimmune diseases.^17^ Insights, benefits, and setbacks of monoclonal antibody therapies have also been presented based on various trials.^18^

Another type of antibody therapy is the use of intravenous infusion of human polyclonal immunoglobulin gamma (IVIG) for treatment. This has been used for the treatment of Kawasaki disease, which was reported as early as in 1950. The pathogenesis of Kawasaki disease is not clear, but is currently listed as an autoimmune disease,^19^ which predominantly affects children <5 years of age and is characterized by systemic inflammation in all medium-sized arteries. However, there are still a number of non-responsive patients under IVIG treatment. Increase in the dosage is not recommended, as high-dose IVIG is strongly associated with hemolytic anemia,^20^ Recently, IVIG plus infliximab (monoclonal antibody against TNF) was used versus IVIG alone to evaluate the effects of combination therapy.^21^ In addition, IVIG was further used as an effective treatment for acute and chronic inflammatory neuropathies, Guillain–Barré syndrome (GBS), chronic inflammatory demyelinating polyneuropathy (CIDP), and multifocal motor neuropathy (MMN). Though IVIG therapy is relatively safe, serious immediate reactions such as anaphylaxis, and delayed complications including thromboembolic events and acute kidney injury may rarely occur.^22^

The rapid emergence of drug-resistant microbes, and newly emerging infectious diseases, together with the ever- increasing number of difficult-to-treat persistent infections, have shown an urgent need for the development of effective preventive and therapeutic antibodies for infectious diseases. Previously, a number of preventive and therapeutic polyclonal and monoclonal antibodies have been under research in academic laboratories, aimed at viral infections caused by rotavirus, Hantaan virus, parvovirus, yellow fever virus.^23^ To date, a number of polyclonal antibodies or immunoglobulins versus hepatitis B virus (HBV), hepatitis C virus, varicella zoster virus, RSV and cytomegalovirus, West Nile Virus and human immunodeficiency virus (HIV) are already being used in diverse applications. Monoclonal antibodies with high neutralizing potency, especially broadly neutralizing antibodies against these infectious agents have been explored. However, their preventive or therapeutic efficacies need further improvement. Importantly, monoclonal antibodies against infections can have a great impact in increasing our capability for rapid response to the public health challenges presented by newly emerging and re-emerging infectious diseases. Indications for the use of antibody therapies in endemic or epidemic of infectious disease have been advised, for treatment of infected individuals, for targeted prophylaxis to protect high-risk individuals, and for targeted prophylaxis to interrupt transmission to average-risk populations.^24^ During the 2014–2016 Zaire ebola virus outbreak in West Africa, ZMapp, a “cocktail” of three mouse–human chimeric antibodies, showed efficacy in nonhuman primates, but it has not been used in humans.^24^ In a recent outbreak of Ebola in Guinea, pooled convalescent plasma was used as an emergency treatment without identifying the neutralizing titer. Totally 84 patients received two consecutive transfusions of 200–250 ml of ABO-compatible convalescent plasma.^25^ Though results showed no improvement in survival, the limited data showed that under outbreaks of lethal infections, antiviral antibody therapies may be considered.

## The crucial roles of Fc and Fc receptors

Neutralizing antibodies of all types play an essential part in antiviral immunity and are instrumental in preventing or modulating viral diseases. Neutralization occurs when the process of virions binding to the cell surface receptors is inhibited or when the fusion processes of virion with cellular endosomal or plasma membranes is disrupted.^26^ Neutralizing antibodies precisely target specific antigens require high levels of affinity maturation. The affinity maturation of antibodies is through multiple rounds of somatic hypermutation and selection in the germinal center.^27^ In addition to directly interfering with virus entry into cells, antibodies can further counteract viral infection through their Fc fragments, triggering immune regulatory mechanisms. These mechanisms have been described as ADCC, antibody-dependent cellular pathogenicity (ADCP), and CDC. ADCC and ADCP activities are mediated by macrophages and natural killer (NK) cells,^28^ whereas CDC is mediated by complement cascade proteins such as C1q and C5 acting on virus-infected cells.^29^

The presence of Fc receptors on the surface of cells of the immune system was reported in late 20th century, and these receptors were subsequently further characterized.^30^ With the identification and cloning of various Fc receptors, the crucial roles of Fc and Fc receptors in therapeutic antibody effects took place and exploitation began. In mice, IgG receptors comprise FcγRI, IIB, III, and IV, whereas in the human counterparts; they are FcγRIA, FcγRIIA, FcγRIIB, FcγRIIC, FcγRIIIA, and FcγRIIIB. Both mouse and human FcγRIIBs contain an intracellular immunoreceptor tyrosine-based inhibitory motif signaling domain and are considered to be inhibitory, whereas all other murine and human FcγRs transduce an activating signal via an immunoreceptor tyrosine-based activation motif (ITAM). In mice, IgG1, IgG2a (IgG2c in C57BL/6 mice), IgG2b, and IgG3, and in human, IgG1-4 are the respective ligands to these receptors. In the absence of crosslinking, all FcγRs are incapable of signaling, except for FcγIR. FcγIR has an extra immunoglobulin domain that permits the highest affinity and binding of the IgG monomer without involving receptor crosslinking. These FcγRs are grouped as type I receptors, whereas type II receptors (DC-SIGN and CD23) are lectin-based Fc receptors that are sensitive to the glycosylation state of Fc. Each receptor family can initiate distinct effector and immunomodulatory pathways.^31^ The conformational diversity of the IgG Fc domain enables a general strategy for shifting receptor specificity that results in different immunological outcomes. When exposed to antigens, specific IgG antibody interaction results in the formation of ICs. The specific composition of IgG subclasses and Fc glycans in ICs determines the degree to which type I or type II FcRs are engaged. Subsequently, effector cells and B cells, in turn, modulate humoral immune processes and innate immune processes.^31^

A number of sugar moieties, such as fucose, galactose, and sialic acid, can be attached to Fc fragments of antibodies.^32^ The immune-mediated effector functions of antibodies are greatly influenced by the Fc glycosylation pattern. For instance, the presence of sialic acid allows changes in the conformation of the heavy chain leading to preferential binding to type II FcγRs. Therefore, this switch in binding affinity is immune regulatory and is in general considered as inhibitory.^31^ Similarly, decrease in core fucose levels can lead to increased affinity of IgG1 for FcγRIII on immune cells, whereas lack of core fucose promotes ADCP.^33–35^ Notably, the Fc glycan composition of human Ig was found to be neither stable nor conserved. The particular Fc glycan composition found in chronic inflammation and in malignancy has been reported to be associated with disease severity and prognosis.^36^ The serum IgG oligosaccharide chains from 22 cancer patients (11 localized cancer and 11 metastatic cancer) and 10 healthy controls have been analyzed. Results showed that serum IgG oligosaccharide chains without galactose were significantly associated with increased tumor progression of lung and gastric cancers.^37^ Another study in 1229 colorectal cancer (CRC) patients showed that plasma IgG glycans correlated with survival outcomes.^36^ Decreased galactosylation, decreased sialylation, and increased bisecting GlcNAc in IgG glycan structures were strongly associated with all-cause (*q* < 0.01) and CRC mortality. Dynamic regulation of glycosylation of Fc has also been observed in pregnancy,^38^ and in the course of treatment of diseases and in vaccination.^39,40^

In addition to engaging classical Fc receptors on the cell membrane, antibodies exert potent immune functions from inside cells via a unique cytosolic receptor for IgG Fc, which is called TRIM21.^41^ The recognition of intracellular antibodies by TRIM21 is of importance for understanding another function of antibodies in responses against microbial infections. Once DNA or RNA non-enveloped virus or intracellular bacteria coated with antibodies enter into cells, TRIM21 is bound and stimulated, which works together with ubiquitin enzymes to target the antibody-coated microbes for destruction via the cellular waste disposal system. At the same time, TRIM21 sends a signal to the cell nucleus to activate certain genes that protect the cell from subsequent infection.^41^ Recently, it was reported that intracellular antibody signaling is regulated by phosphorylation of TRIM21, and the activation of TRIM21 is independent of all known pattern recognition receptors (PRRs).^42^ Activation of TRIMK21 catalyzes K63-ubiquitin chain formation, thereby stimulating transcription factor pathways involving NF-κB, AP-1 and IRF3, IRF5, IRF7. The activation of these factors results in proinflammatory cytokine production.^43^ The genes activated by TRIM21 have potent antiviral activity. However, if they are wrongly switched on, autoimmune diseases like rheumatoid arthritis and multiple sclerosis may occur. It is still unknown how TRIM21 is precisely regulated and only activated during an infection. Another receptor in the FcR family is neonatal FcR (FcRn), which is an Major Histocompatible Complex (MHC) class I-related protein associated with β2m. This endosomal protein binds to IgG in low pH environments (pH < 6.5) and plays a role as IgG transporter in epithelial/endothelial cells. In addition to IgA, which can be secreted at mucosal sites, FcRn can also be used as a tool to target antigens to mucosal sites.^44^

## Antigen–antibody ICs: a double-edged sword

Antigen–antibody ICs can either cause immune pathological effects or potentiate beneficial immune effects, depending on various factors, including the subclasses of the antibody, the ratio between the antigen and the antibody forming the IC, the biological characteristics of the IC components, the sites where the ICs were formed, the cells involved and how the ICs were introduced into hosts etc. Table 1 shows comparisons between ICs causing pathological outcomes versus ICs inducing immune regulatory effects.

**Table 1:** Comparison of ICs exerting immune regulatory versus immune pathological functions

	Immune regulatory functions	Pathological functions	References
Components	Ag and high-affinity Ab	Ag and low-affinity Ab	^27^
Deposit on vessels/binding to FcγR	– Ag:Ab in circulation; – Size, subclasses, glycosylation of IgG determine IC binding to FcγR;	– Ag:Ab cannot be cleared by phagocytosis; – Deposited on blood vessels and organs;	^30, 45^
Immunological outcomes	– Interact with DC, enhance Ag presentation; – Induce effective T-cell-mediated immune responses; – Induce high titer broadly reactive antibodies; – Reshape intracellular responses;	– Trigger ADCC, ADCP, CPC; – Intrinsic ADE; – Activate CD16^+^ monocyte (SLE); – Inflammation; – Pathological lesions;	^29, 47, 48, 55– 58, 63, 64, 66, 99^
Biological/medical implementation	Generate broadly neutralizing antibodies, develop new vaccines	New targets and drug development	^79, 86, 91^

With the evolving progress of using therapeutic antibodies or immunoglobulins for treatment, in recent years, the immunopathological effects of ICs alongside with their therapeutic efficacies have been studied in depth. The conventional concept of IC-mediated immunological pathogenesis has been that, when ICs were not cleared by phagocytosis system, they remained in blood circulation and deposited on small vessel walls of various organs. These deposited ICs could exert damaging effects by binding to complement receptors on innate immune effector cells and result in inflammation and tissue injury. However, through studies on soluble ICs and their effects, it was observed that the fate of ICs in blood circulation is either to initiate immunopathological outcomes, or to react with receptors on immune cells initiating immunological regulations. Decreased binding of ICs to Fc receptors could affect biological outcomes. In a study to analyze elements involved in ICs binding to Fc receptors, the size of IC, IgG subclasses, glycosylation of IgG, all were found of relevance.^45^ Mechanistic study of the pathological injuries in arthritis patients and IC-induced nephritis revealed that binding of ICs to FcγRI (CD64) contributed to the severity of arthritis and hypersensitivity responses.^46^ In lupus nephritis, intra-capillary IC deposits selectively accumulated a proinflammatory population of 6-sulfo LacNAc+ (slan) monocytes (slanMo), which locally expressed TNF-α.^47^ The recruitment of slanMo from the microcirculation was via interaction with Fc γ receptor IIIA (CD16) and the slanMo then induced the production of neutrophil-attracting chemokine CXCL2, as well as TNF-α.

In microbial infections, more pathogenic mechanisms have been described. When ICs formed between non-neutralizing IgG and microorganisms that can replicate in macrophages, increased intracellular infections can occur and this was named intrinsic antibody-dependent enhancement (ADE) of infections.^48^ This ADE of infection modulates the severity of diseases such as dengue hemorrhagic fever and leishmaniasis. Intrinsic ADE is distinct from extrinsic ADE, because intrinsic ADE leads to an increased number of infected cells.^48^ The mechanism manifests as suppression of host innate immunity through idiosyncratic Fcγ, increased production of IL-10, a bias of Th1 responses towards Th2 responses and increased numbers of infected cells.

Recently, another new immune inhibitory mechanism of ICs was reported in a mouse model of persistent lymphocytic choriomeningitis virus (LCMV) infection.^49^ The increased amounts of IC in the circulation during persistent infections, competed with FcγR binding and suppressed multiple aspects of FcγRs-dependent responses in vivo. The FcγR-mediated processes that were suppressed in vivo included activation of innate cells such as NK cells. By using transgenic mice expressing human CD20 and chronically infected with LCMV, virus antibody IC in circulation was shown to hamper the depletion of B cells by an anti-CD20 antibody (rituximab), a drug for treatment of B-cell lymphoma. In addition, FcR-dependent activation of dendritic cells by agonistic ant-CD40 antibody was decreased by the persistence of IC in these mice.^50^ Though these findings are not directly associated with IC pathogenicity, the data suggest that ICs could limit the effectiveness of therapeutic antibodies in humans.

Consistent with the role of ICs as a double-edged sword, ICs have shown immune regulatory functions that potentiate or restore favorable immune responses. Long before the discovery of Fc receptors, Terres et al. observed that when antibody was combined with its antigen at an appropriate ratio, IC could enhance antibody response in animals.^51^ Later, the potentiating effects of IC were shown with structural protein and antibody to paramyxovirus Simian virus 5,^52^ with hepatitis B surface antigen (HBsAg) and its antibodies^53^ and with antibody to HIV in an in vitro study with peptides.^54^

Following the discovery of Fcγ receptors, mechanistic studies on how ICs potentiate immune responses progressed with focus on IC–cell interactions. Hamano et al. showed that the efficient priming of Th cell responses by APCs in vivo was IC dependent.^55^ In cancer antitumor vaccine studies, IC-loaded dendritic cells (DCs) were found superior to soluble ICs.^56^ Circulating antibodies were shown to enhance systemic cross priming by delivery of antigens to DCs^57^ and ICs not only induced DC maturation in vitro, but also enabled DCs to prime peptide-specific CD_8_^+^ CTLs in vivo.^58^ These dual roles in enhancement of Ag uptake and activation of DCs and in priming of CD_8_^+^ CTL responses to exogenous antigens, resulted in a “license to kill” function. In experimental studies, formation of complexes of cellular antigen with antibody resulted in activation of dendritic cells, facilitation of cross-presentation of antigens to tumor-specific CD_8_^+^ T cells and inhibition of tumor growth Fc receptor-targeted antigen uptake was shown to initiate cross-presentation pathways as an immune regulatory mechanism for effective tumor immunity. In consequence, Fc receptor targeting was considered in tumor vaccine development.^59^ ICs potentiation effect on B cells was shown to be mediated by ICs retention in follicular dendritic cells (FDCs), and reappearance on the cell surface, thereby becoming available to B cells.^60,61^

Immune regulatory effects of ICs have been shown to restore effective immune responses against infections. In a mouse model of persistent infection, IL-2/anti-IL-2 (IL-2 IC) was shown to increase the numbers of virus-specific CD_8_^+^ T cells and enhance cytotoxicity mediated by the perforin–granzyme pathway.^62^ Optimized adenovirus–antibody complexes were shown to stimulate strong cellular and humoral immune responses via a significantly extended duration of antigen availability and enhanced lymphocyte activation kinetics. Formation of IC with antibody and rabies virus G antigen on cell surface redirected the native intracellular pathway,^63^ suggesting that some new immunoregulating mechanisms might be generated by viral ICs in cells. Furthermore, study of the functions of dendritic cells carrying IC showed prolonged presentation of antigen. This effect was virus specific and was dependent on a switch of antibody isotypes.^64^

The use of new technologies has enabled new progress on immune regulatory functions associated with glycosylation profiles of IgG and detailed studies on IC-induced immunoregulatory pathways. The uptake of IC after ligation activated FcγR on DC, leading to 100 times more antigen presentation than uptake of free antigen. The activated FcγRs elicited signaling via the ITAM domain of the FcγR chain.^65^ Splenic DCs from NOTAM mice were used to identify the role of ITAM domain signaling in cross-presentation of soluble IC by DCs. Results showed that signaling by the ITAM domain of FcγR chain was critically required for IC presentation, but not for MHC class II antigen presentation.^65^ In a study to reveal the immunological mechanisms leading to the development of HIV broadly neutralizing antibodies, differences in IC biology in a group of spontaneous controllers of HIV (≤2000 copies/ml) were identified in comparison with normal progressors. Polyclonal ICs and monoclonal IC from neutralizers were more effective than those from progressors in inducing higher antibody titers, higher-avidity antibodies, and expanded DC–B-cell reactions after immunization of mice.^66^ The results implicated altered Fc profile/complement interactions exerted differentially shaping the maturation of the humoral immune response. It was speculated that the enhanced Fc functions could actively contribute to the evolution of a broader HIV-specific neutralization range.^66^

**Fig. 1:**
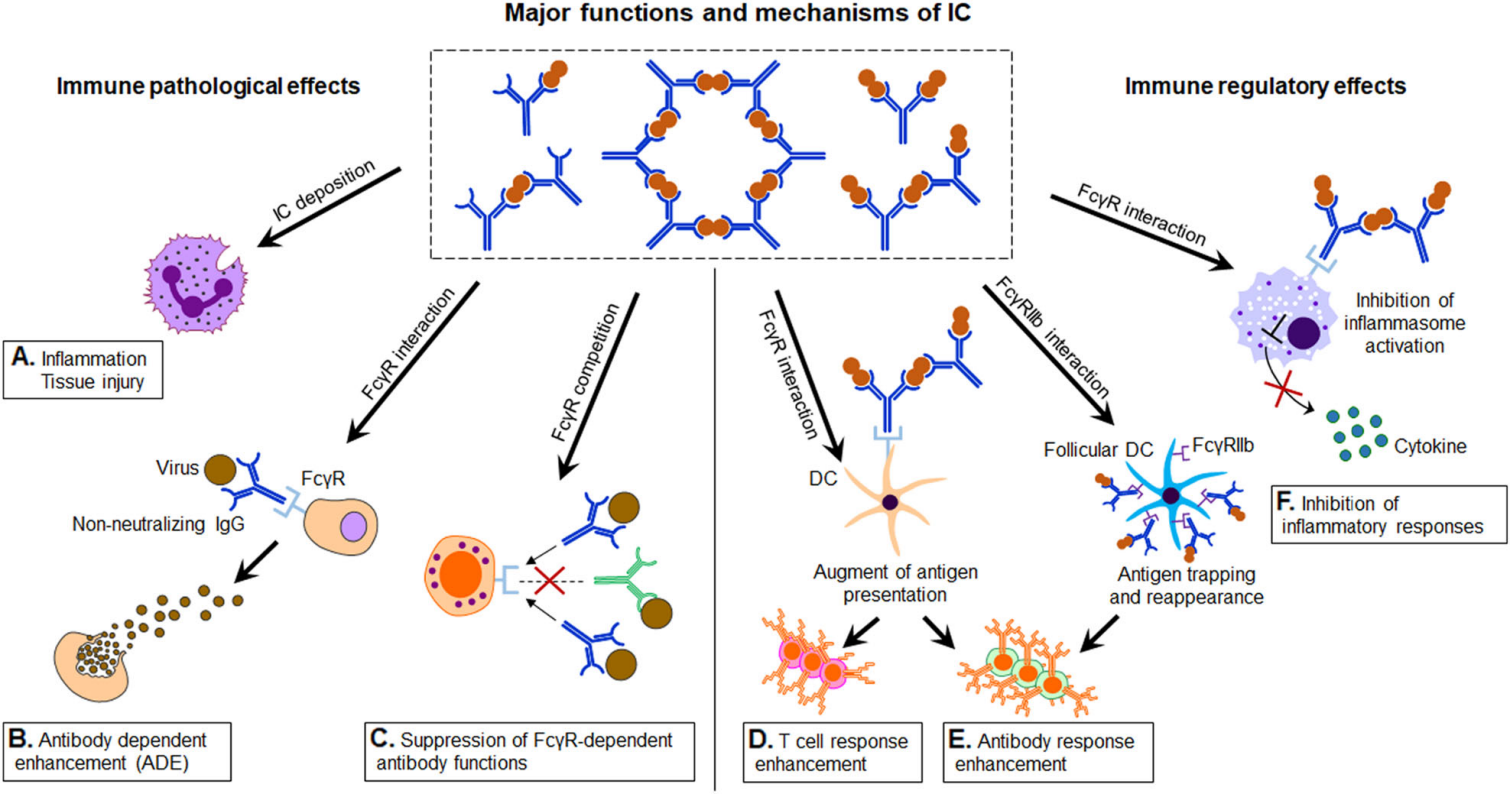
The immune regulatory functions of immune complex (IC) in therapy and vaccine. Summary of the major functions and mechanisms of ICs, showing the immune pathological effects and immune regulatory effects. The blue “Y” shape figure represents antibody, and the red round represents antigen. Immune pathological effects (**a** inflammation, tissue injury; **b** antibody-dependent enhancement; **c** suppression of FcγR-dependent antibody functions) and immune regulatory effects (**d** T-cell response enhancement; **e** antibody response enhancement; **f** inhibition of inflammatory responses)

In addition to their immune regulatory functions, ICs can effectively inhibit inflammatory responses. ICs were shown to inhibit the adaptive immune responses in an NLRP3-dependent model during priming of immune responses in vivo,^67^ suppression of both inflammasome activation and the generation of IL-1 alpha and IL-1 beta from antigen-presenting cells were observed. Recently, IL-2/IL-2 antibody IC was found to regulate HSV-induced inflammation through induction of IL-2 receptors alpha, beta, and gamma in a mouse model.^68^ The anti-inflammatory function has been widely employed in therapeutics for various diseases. A favored approach has been to use ICs in combination with cytokines and their antibodies. IL-2 complex treatment expanded both the NK and CD_8_^+^ T memory cell pool, including preexisting memory-phenotype T cells. In a renal ischemia–reperfusion injury (IRI) mouse model, IL-2 IC reduced expression of inflammatory cytokines and attenuated the infiltration of neutrophils and macrophages in renal tissue.^69^ IL-2 IC treatment has also been studied in experimental renal cancer.^69^ In experimental atherosclerosis, IL-2 IC in combination with anti-CD3 antibody markedly reduced atherosclerosis lesions.^70^ This effect was accompanied by a striking increase in the Treg/Teff ratio in the T cells in lymphoid organs and atherosclerotic lesions. Naive mice treated with a short course of IL-2 complexes showed enhanced protection from newly encountered bacterial and viral infections.^71^ However, increased IL-2 complex treatment generated CD_8_^+^ T cells and NK cells with a reduced capacity to produce IFN-γ, potentially suggesting some form of exhaustion occurred. Figure 1 summarizes the various known immunological functions of ICs (Fig. 1).

## Preventive and therapeutic vaccines based on IC

The content of this section has been published in a previous review,^72^ herein a short summary of IC vaccines and new developments is presented.

The first application of an IC vaccine was in the prevention of infectious bursa disease (IBD) in poultry. This viral infection targets the bursa of Fabricius and kill developing B cells. More recently, in an in ovo application of the IC vaccine against Newcastle Disease Virus (NDV) in maternal antibody-positive chickens, the birds were protected against clinical disease. An IC-based vaccine has also been used to protect pigs against pig parvovirus infections. This vaccine was shown to be both safe and ecologically convenient.

The first glimmer of success with an IC vaccine in HIV infections was reported by Hioe et al., who demonstrated that gp120 antigenicity and immunogenicity were significantly enhanced when gp120 was presented as an IC with anti-CD_4_ mAb 654-D. This enhanced the antigenicity and immunogenicity of gp120s from different HIV-1 strains and elicited neutralizing antibodies in mice.^73^ Later, Gp120/654 complex was shown to not only induce anti-gp120 antibodies to higher titers, they were also cross-reactive with V3 peptides from most subtype B and some subtype C isolates.^74^ Recently, a prime/boost immunization strategy was shown to facilitate Fc-mediated phagocytosis. Another research group used an SIV model to explore an IC vaccine as a topical preventive vaccine for women.^75^ They showed that an Simian Immunodeficient Virus (SIV) -specific IC could interact with the FcγRIIb receptor on the epithelium lining the cervix and block target cell recruitment.

Broadly protective/universal vaccines that can provide immunity against seasonal influenza virus strains and potential pandemic viruses have been explored. Recently, to define the influence of the sialylation of the Fc glycan on the effect of Fc–FcγR interaction, novel IC vaccines have been studied. A seasonal trivalent influenza vaccine (TIV) IC was constructed using hemagglutinin (HA) complexed with polyclonal human IgG or with mAb PY102 IgG:HA trimer. Immunization of healthy adults showed that the abundance of sialyated Fc (sFc) on the anti-HA IgGs affected induction of the early plasmablast response and correlated with vaccine efficacy.^76^ In mice, TIV-sFc induced significantly higher titers of antibodies and increased binding efficiency to the H1 from several other strains, and to H3 and even H5 from avian influenza virus.^76^ The mechanisms involved were an engagement of germinal center B-cell CD23 by sFc within the HA ICs, an upregulation of FcγRIIb, and modulation of the selection of B cells in favor of those expressing higher-affinity B-cell receptors.^77^

The global prevalence of chronic HBV infection (CHB) is estimated to be around 250 million. Defects in cell-mediated immune responses and immune tolerance towards HBV are the key issues for chronicity. Restoration of effective cell-mediated immune responses has been explored in different Immunotherapeutic approaches.^78^ A recent review presented approaches to developing more effective therapeutic vaccines,^79^ suggesting to use more potent immunogens that can stimulate both T- and B-cell responses, developing a better prime and boost strategy, or employing immune checkpoint inhibitors in combination therapy.

The development of HBsAg-HBIG IC as a therapeutic vaccine for CHB was based on the concept that the Fc fragment of antibodies in the IC could interact with Fc receptors on DCs cells, and initiate more effective immune responses in hosts.^80^ This vaccine has been evaluated by in vitro experiments,^81^ in HBsAg-positive transgenic mice,^82^ and finally in clinical trials.^83,84^ By live-cell imaging technology in vitro, immune-potentiated pathways of IC internalization and dissociation of IC in cells were kinetically followed.^85^ After IC internalization by antigen-presenting cells, IC was subsequently transferred through early and late endosomal into lysosomal compartments. Dissociation of IC into antigen and antibody was mainly observed in the late endosome.^85^

Data from clinical trials, for the first time, provided important information regarding responses in normal adult and chronic hepatitis patients immunized with IC. The therapeutic efficacies of this IC were similar to those of interferon-α treatment and the protocol for treatment was deemed to merit further optimization^83^ Importantly, transient flares of alanine aminotransferase (ALT), reflecting injuries in liver function, were observed in a small percentages of IC-treated patients, but liver functions became normal during treatment or at the follow-up period. Interestingly, most of the patients with flares in liver function were responsive to IC immunization.^81,83^ This observation suggested that immune responses induced by IC played dual roles, the enhanced immune responses not only targeted the virus but also targeted host hepatocytes harboring the virus. Most likely, T cytotoxic cells or NK cells were transiently “turned on” in responsive patients, but this response was reversible. To further explore therapeutic mechanisms of IC in patients, a recent clinical study on IC immunization was done using IC co-administered with an anti-HBV drug (adefovir) and using alum and normal saline as controls. Cytokines from peripheral blood mononuclear cells (PBMCs) stimulated with HBV-specific peptides were assayed and analyzed. In the IC-immunized group of patients, increases in Th1 and Th2 cells among the CD_4_^+^ T cells were associated with decrease in Treg cells and increase in Tc1 and Tc17 cells among the CD_8_^+^ T cells.^86^

Subcutaneous immunization of Balb/C mice with purified (Ebola immune complex) EIC resulted in anti-Ebola virus antibody production at levels similar to those obtained with a GP1 virus-like particle.^87^ The results indicated excellent potential for using a plant-expressed EIC as a human vaccine. Adenovirus–antibody complexes have promoted cellular and humoral responses in naive individuals in addition to those with preexisting immunity. IC has stimulated effective immune responses against the highly infectious disease caused by *Francisella.tularensis* (Ft), a category A biothreat agent. Recently, without exogenous adjuvants, a hybrid dengue-Ebola recombinant IC (DERIC) induced a potent, dengue virus-neutralizing anti-cEDIII humoral immune response in mice. This potential basis for a universal RIC platform for other antigens awaits confirmation in field trials.^88,89^

The main obstacles for the development of therapeutic cancer vaccines are: tumor evasion from recognition by the host immune system, tumor inhibition of immune responses, and defective induction of adaptive immunity.^90^ In experimental studies, antitumor monoclonal antibody could generate antibody–tumor antigen ICs to initiate host immune responses.^59^ and IC-loaded DCs were shown to be superior to soluble ICs in tumor immunotherapy.^56^ More recently, scientists demonstrated that antibody–tumor antigen ICs engaged the hFcγRIIIA expressed by phagocytes to initiate ADCC, and engage the hFcγRIIA to stimulate DC maturation and presentation of tumor antigens to T cells.^91^ Despite all these excellent experimental studies, no IC cancer vaccine is currently under clinical trial. One potential candidate IC for cancer patients is IL-2-anti-IL-2 complex (IL-2 IC), which could extend IL-2 bioactivity from hours to days.^92^ Furthermore, the antibody component in the IC can be manipulated to interact with specific cellular receptors, focusing IL-2 towards specific cells such as CD_8_^+^ T cells, NK cells, and Treg cells.

## Perspectives

ICs are major mediators of regulated immune responses and have been extensively studied in experiments, in vitro and in vivo. The uniqueness of ICs is that they are generated naturally in hosts via interactions between the products of humoral immune responses and their respective antigens. Antigen–antibody IC-based vaccines mimic natural IC functions in experimental animals. Importantly, the effects of ICs are not limited to modulating humoral responses, shown by IC–DC interactions, but also include the initiation of a range of immune responses. With different compositions of antibody Fcγ and interactions with different types of FcγR, ICs can exert multiple functions, resulting in modulation or reshaping of diverse immune responses. In consequence, ICs are under experimental and clinical studies for the prevention and treatment of diverse diseases.^93,94^

Previously, a major handicap for developing IC vaccines for human use was the lack of appropriate human monoclonal antibodies for the construction of IC vaccines that would function properly through human Fc receptors. With the rapid development and production of different human monoclonal antibodies for therapeutic purposes, switching from polyclonal human immunoglobulin to specific human monoclonal antibody may significantly improve the efficacies of IC therapies. In addition, the use of ICs with modifications in the glycosylation of IgG can be employed to generate broadly neutralizing antibodies for protection against viruses that are prone to mutate.

With the experience gained in producing ICs with HBsAg and influenza HA and their respective antibodies, appropriate ratios and methodologies for the manufacture of ICs can be standardized to fulfill the regulatory requirements for clinical application. Furthermore, the recent development of a simple cellular assay of IC-mediated T-cell activation in vitro using human peripheral blood mononuclear cells, may help to evaluate the efficacy of ICs prior to clinical trials.^95^ Notably, due to the small dosage of monoclonal antibodies used to generate ICs, it will also be intrinsically less expensive to produce ICs than therapeutic antibodies. Furthermore, the tedious and expensive process of separately producing qualified antigen and antibody may be avoidable. Recombinant ICs can also be generated by fusing antigens with the Fc fragment of IgG into one molecule and expressing the construct in appropriate vectors.^96^ This experimental approach has been tested using plant biotechnology and immunization in mice.^87^ In addition, as shown in a recent study on allosteric communications in antibody–antigen recognition and FcR activation,^97^ more effective IC constructs may be generated.

Preventive IC vaccines have certain advantages over the traditional preventive vaccines. However, due to the scale of the existing industrial commitment to producing the established kinds of preventive vaccines, application of preventive ICs may be limited to a few vaccine targets. In contrast, therapeutic IC vaccines seem to have a broader opportunity in practice. Combination therapy is the current trend for the cure of diseases and therapeutic IC vaccines may be given in combination with drugs, or other immune regulatory factors, thereby achieving better therapeutic efficacy than either of the treatments alone. Such a sandwich strategy using IC in combination with antiviral drugs and antibodies has been suggested.^98^

Although extensive experimental studies have shown the immune regulatory effects of ICs, the application of ICs in vaccinology has only just started. As IC therapy is mediated through immune regulation, and immune responses initiated by IC can be pathological, as shown by transient elevation of ALT in certain IC-treated CHB patients. Careful monitoring of side effects during IC clinical trials is crucial. More field/clinical trials are clearly merited to finally substantiate and verify ICs’ contribution to vaccinology.
